# Meta-analysis of randomized trials: evaluation of benefit from gemcitabine-based combination chemotherapy applied in advanced pancreatic cancer

**DOI:** 10.1186/1471-2407-8-82

**Published:** 2008-03-28

**Authors:** Volker Heinemann, Stefan Boeck, Axel Hinke, Roberto Labianca, Christophe Louvet

**Affiliations:** 1 Department of Internal Medicine III, Klinikum Grosshadern, University of Munich, Germany; 2 WiSP, Langenfeld, Germany; 3 Ospedali Riuniti, Bergamo, Italy; 4 Service d'Oncologie, Médecine Interne, Hôpital St. Antoine, Paris, France

## Abstract

**Background:**

Single-agent gemcitabine (GEM) is a standard treatment for advanced and metastatic pancreatic cancer. This study examines the question whether GEM-based combination chemotherapy can further improve treatment efficacy.

**Methods:**

A meta-analysis was performed to evaluate randomized trials comparing GEM versus GEM+X (X = cytotoxic agent). Fifteen trials including 4465 patients were eligible for an analysis of overall survival, the primary end-point of this investigation.

**Results:**

The meta-analysis revealed a significant survival benefit for GEM+X with a pooled hazard ratio (HR) of 0.91 (95% CI: 0.85 – 0.97, p = 0.004). The overall test for heterogeneity resulted in p = 0.82 (I^2 ^= 0%). The analysis of platinum-based combinations indicated a HR of 0.85 (95% CI: 0.76 – 0.96, p = 0.010), while for fluoropyrimidine-based combinations the HR was 0.90 (95% CI: 0.81 – 0.99, p = 0.030). No risk reduction was observed in the group of trials combining GEM with irinotecan, exatecan or pemetrexed (HR = 0.99). A meta-analysis of the trials with adequate information on baseline performance status (PS) was performed in five trials with 1682 patients. This analysis indicated that patients with a good PS had a marked survival benefit when receiving combination chemotherapy (HR = 0.76; 95% CI: 0.67 – 0.87; p < 0.0001). By contrast, application of combination chemotherapy to patients with an initially poor PS appeared to be ineffective (HR = 1.08; 95% CI: 0.90 – 1.29, p = 0.40).

**Conclusion:**

The meta-analysis of randomized trials indicated a significant survival benefit when GEM was either combined with platinum analogs or fluoropyrimidines. Based on a preliminary subgroup analysis (representing 38% of all patients included in this meta-analysis), pancreatic cancer patients with a good PS appear to benefit from GEM-based cytotoxic combinations, whereas patients with a poor PS seem to have no survival benefit from combination chemotherapy.

## Background

Pancreatic cancer is the fourth or fifth leading cause of solid tumour deaths in Western industrialized countries. Due to its predominantly late diagnosis, most patients are diagnosed with advanced or metastatic disease at first presentation [1,2]. Without effective treatment, a median survival of only 3 to 4 months is expected in metastatic disease.

Single-agent gemcitabine has evolved as a standard of care for treatment of locally advanced and metastatic pancreatic cancer. However, treatment effects remain moderate with median overall survival (OS) times in the range of 5 to 8 months and 1-year survival rates in the range of 17–25%.

To improve therapeutic efficacy numerous randomized trials have investigated gemcitabine-based combination regimens adding a second cytotoxic agent such as a platinum analog [3-7], a fluoropyrimidine [8-13], a multitarget antifolate [14] or topoisomerase inhibitors [15-17]. While some studies showed an improvement of objective response rates (ORR) and progression-free survival (PFS) with combination chemotherapy, most trials lacked statistical power and failed to demonstrate a statistically significant prolongation of survival. So far, only the preliminary results of one randomized study showed a significant survival benefit in favour of combination chemotherapy [13].

The present analysis tries to overcome the statistical limitations of the individual trials and investigates the treatment effects in total and in various combination groups. Three groups characterized by the combination partner were formed prospectively: in the first group, gemcitabine was combined with a platinum analog like oxaliplatin or cisplatin. The second group included fluoropyrimidines like 5-fluorouracil (5-FU) or capecitabine as combination partners; the third group comprised all other cytotoxic agents such as pemetrexed, irinotecan, and exatecan.

## Methods

The meta-analysis was performed according to a prospectively written protocol and analysis plan.

### Selection of trials

Trial selection was performed independently by three of the authors (V. H., S. B. and A. H.); trial quality evaluation was done by A. H. Randomized trials were selected for evaluation when they investigated the first-line chemotherapy of histologically confirmed locally advanced or metastatic pancreatic cancer. As a consequence, all studies performed in the adjuvant or neoadjuvant setting were excluded. Only those studies entered the analysis which used single-agent gemcitabine in the control arm and gemcitabine-based two-drug combination chemotherapy in the experimental arm. The availability of adequate survival data was an inclusion criterion for the selected randomized phase II and phase III studies. Since the analysis was confined to the evaluation of chemotherapeutic agents only, trials investigating targeted agents such as metalloproteinase inhibitors, tipifarnib, erlotinib, bevacizumab or cetuximab were not included.

### Search for trials

Trials were included into the analysis which had been published until the year 2006. The *PubMed *database was searched for publications related to the use of chemotherapy in advanced pancreatic cancer. In addition to full publications, abstracts presented at the annual meetings of the American Society of Clinical Oncology (ASCO) and the European Cancer Conference (ECCO) were also included. The search was performed using the following terms: "pancreatic cancer", "chemotherapy", "randomized controlled trial". Moreover, information from medical experts and pharmaceutical industry on additional relevant data was retrieved.

### Assessment of validity

An open assessment of the trials was performed according to Jadad and coworkers [18].

### Data abstraction

Data abstraction was performed by two independent observers who extracted the data from the respective trials and verified the results by comparison.

### Statistical methods

Individual patient data were available in two trials only, and were the preferred source for analysis in these cases. The data from the other studies could be retrieved from peer-review publications of 8 trials, while the remaining 5 trials were only recently analysed, providing the required information in abstracts and presentation slides/posters. Extraction of summary statistics from the published data was performed according to standard methods for survival endpoints [19]. Standard techniques for meta-analysis were used [20], as incorporated in the software packages METASUB V. 1.1 (idv, Gauting, Germany) and Review Manager V. 4.2 (Nordic Cochran Centre, Copenhagen). Both fixed (primarily) and random effect model methodology was applied. All reported p-values result from two-sided versions of the respective tests. The revision of funnel plots did not reveal any indications of major publication bias.

## Results

### Characteristics of the 15 randomized trials of the meta-analysis

This meta-analysis evaluated 4465 patients in 15 randomized trials, of whom 2243 patients were included into the control arm and 2222 patients into the combination arm. One additional trial including 42 patients fulfilled the selection criteria, but had to be excluded, as information was available only as abstract and insufficient for appropriate survival hazard analysis [21]. Single-agent gemcitabine was generally applied in the control arms: ten trials [3,4,6,7,9,10,12,13,15,17] used the gemcitabine regimen introduced by Burris et al where gemcitabine was given at a dose of 1000 mg/m^2 ^(either as 30-minute infusion or as fixed-dose rate (FDR) infusion with 10 mg/m^2^/min) for seven out of eight weeks, then followed by a weekly drug application for three out of four weeks [22]. In further four trials gemcitabine was given weekly times three every four weeks [5,8,14,16], while in one trial high-dose gemcitabine was applied at 2-week intervals [11].

Baseline characteristics of the individual trials including gender, performance status (ECOG performance status 0–1 or Karnofsky performance status (KPS) 90–100%) and stage of disease (locally advanced versus metastatic) are indicated in Table 1, 2, 3. The distribution of baseline patient characteristics within the respective 15 trials was found to be quite homogeneous. However, between the different trials a considerable degree of variation can be detected. For example, the percentage of patients with metastatic disease ranges from 54% to 100%, while the fraction of good performance status patients varies from 24% to 88%.

**Table 1:** Characteristics of 5 randomized trials comparing gemcitabine to gemcitabine plus platinum analog

**Reference**	**Year**	**n**	**Treatment regimen**	**Stage IV (%)**	**Male (%)**	**PS 0–1 (%)**
Louvet	2005	156	Gem 1000 mg/m^2 ^for 7 of 8 wks, then wkly for 3 of 4 wks	70	53	82
		157	Gem 1000 mg/m^2^/100 min d1 (FDR) + Oxaliplatin 100 mg/m^2 ^d2 q 2 wks	68	60	83
Poplin	2006	279	Gem 1000 mg/m^2 ^for 7 of 8 wks, then wkly for 3 of 4 wks (standard)	88	56	88
		277	Gem 1500 mg/m^2^/150 min q wk × 3 of 4 wks (FDR)		58	
		276	Gem 1000 mg/m^2^/100 min d1 (FDR) + Oxaliplatin 100 mg/m^2 ^d2 q 2 wks		46	
Heinemann	2006	97	Gem 1000 mg/m^2 ^for 3 of 4 wks	79	62	49*
		98	Gem 1000 mg/m^2 ^+ Cisplatin 50 mg/m^2 ^q 2 wks	80	65	56*
Colucci	2002	54	Gem 1000 mg/m^2 ^for 7 of 8 wks, then wkly for 3 of 4 wks	54	50	med. KPS 70
		53	Gem 1000 mg/m^2 ^+ Cisplatin 25 mg/m^2 ^for 6/7 wks	62	66	med. KPS 70
Viret	2004	41	Gem 1000 mg/m^2 ^for 7 of 8 wks, then wkly for 3 of 4 wks	78	na	83
		42	Gem 1000 mg/m^2 ^d1, 8, 15 + Cisplatin 75 mg/m^2 ^d15 q 4 wks	81	na	76

* KPS = 90–100%; PS = performance status; FDR = fixed dose rate; na = data not available;

**Table 2:** Characteristics of 6 randomized trials comparing gemcitabine to gemcitabine plus fluoropyrimidine

**Reference**	**Year**	**n**	**Treatment regimen**	**Stage IV (%)**	**Male (%)**	**PS 0–1 (%)**
Berlin	2002	162	Gem 1000 mg/m^2 ^for 3 of 4 wks	90	54	86
		160	Gem 1000 mg/m^2 ^+ 5-FU 600 mg/m^2 ^for 3 of 4 wks	89	52	86
Riess	2005	238	Gem 1000 mg/m^2 ^for 7 of 8 wks, then wkly for 3 of 4 wks	77	54	48*
		235	Gem 1000 mg/m^2 ^+ FA 200 mg/m^2 ^5-FU 750 mg/m^2 ^CI × 4 wks q 6 wks	77	52	44*
DiCostanzo	2005	48	Gem 1000 mg/m^2 ^for 7 of 8 wks, then wkly for 3 of 4 wks	73	48	69
		43	Gem 1000 mg/m^2 ^+ CI 5-FU 200 mg/m^2 ^for 6 of 7 wks, then wkly for 3 of 4 wks	67	63	67
Scheithauer	2003	42	Gem 2200 mg/m^2 ^q 2 wks	100	55	24
		41	Gem 2200 mg/m^2 ^+ Capecitabine 2500 mg/m^2 ^d1-7 q 2 wks	100	66	27
Herrmann	2005	159	Gem 1000 mg/m^2 ^for 7 of 8 wks, then wkly for 3 of 4 wks	79	53	53*
		160	Gem 1000 mg/m^2 ^d1, 8 + Capecitabine 2 × 650 mg/m^2 ^d1-14 q 3 wks	80	54	53*
Cunningham	2005	266	Gem 1000 mg/m^2 ^for 7 of 8 wks, then wkly for 3 of 4 wks	71	na	82
		267	Gem 1000 mg/m^2 ^d1, 8, 15 + Capecitabine 2 × 830 mg/m^2 ^d1-21 q 4 wks	70	na	81

* KPS = 90–100%; PS = performance status; na = data not available;

**Table 3:** Characteristics of 4 randomized trials comparing gemcitabine to gemcitabine plus other cytotoxic agents

**Reference**	**Year**	**n**	**Treatment regimen**	**Stage IV (%)**	**Male (%)**	**PS 0–1 (%)**
Oettle	2005	282	Gem 1000 mg/m^2 ^for 3 of 4 wks	92	54	88
		283	Gem 1250 mg/m^2 ^d1, 8 + Pemetrexed 500 mg/m^2 ^d8 q 3 wks	90	60	85
Rocha Lima	2004	180	Gem 1000 mg/m^2 ^for 7 of 8 wks, then wkly for 3 of 4 wks	81	53	74
		180	Gem 1000 mg/m^2 ^+ Irinotecan 100 mg/m^2 ^d1+8 q 3 wks	82	57	78
Stathopoulos	2006	70	Gem 900 mg/m^2 ^for 3 of 4 wks	86	60	86
		60	Gem 900 mg/m^2 ^d1, 8 + Irinotecan 300 mg/m^2 ^d8, q 4 wks	78	65	87
O'Reilly	2004	174	Gem 1000 mg/m^2 ^for 7 of 8 wks, then wkly for 3 of 4 wks	78	57	52*
		175	Gem 1000 mg/m^2 ^d1, 8 + Exatecan 2 mg/m^2 ^d1+8 q 3 wks	79	53	51*

* KPS = 90–100%; PS = performance status;

### Gemcitabine plus platinum analog versus single-agent gemcitabine

Five randomized trials compared the combination of gemcitabine plus a platinum analog (n = 623) with gemcitabine alone (n = 625) (Tables 1, 4, 5). They included two oxaliplatin-based and three cisplatin-based combination studies. The platinum-based combinations induced a significant improvement of ORR and PFS in two trials [3,6], while the level of significance was not reached in further three trials [4,5,7]. The platinum-based combination regimens consistently prolonged OS. None of the individual trials showed, however, a statistically significant superiority compared to gemcitabine alone. A significant improvement of OS was detected only when a combined analysis of the five trials was performed (HR = 0.85, p = 0.010).

**Table 4:** Overall response rate and PFS in 5 randomized trials comparing gemcitabine to gemcitabine plus platinum analog

**Reference**	**Year**	**n**	**Treatment regimen**	**ORR (%)**	**p**	**Median PFS/TTP (mo)**	**p**
Louvet	2005	156	Gemcitabine	17.3	0.04	3.7	0.04
		157	Gemcitabine (FDR) + Oxaliplatin	26.8		5.8	
Poplin	2006	279	Gemcitabine (standard)	5	--	na	na
		277	Gemcitabine (FDR)	10	--		
		276	Gemcitabine (FDR) + Oxaliplatin	9	--		
Heinemann	2006	95	Gemcitabine	8.2	--	3.1	0.053
		95	Gemcitabine + Cisplatin	10.2		5.3	
Colucci	2002	54	Gemcitabine	9.2	0.02	2.0	0.048
		53	Gemcitabine + Cisplatin	26.4		5.0	
Viret	2004	41	Gemcitabine	5	--	2.5	ns
		42	Gemcitabine + Cisplatin	7		2.2	

ORR = overall response rate; PFS = progression-free survival; TTP = time-to-progression; FDR = fixed dose rate; na = data not available; ns = not significant;

**Table 5:** Survival in trials comparing gemcitabine to gemcitabine plus platinum analog

**Reference**	**Year**	**Treatment regimen**	**Median survival (mo)**	**p**	**HR**	**95% CI**
Louvet	2005	Gemcitabine	7.1	0.13	0.82	0.64 – 1.05
		Gemcitabine (FDR) + Oxaliplatin	9.0			
Poplin	2006	Gemcitabine (standard)	4.9	--	0.83*	0.69 – 1.00
		Gemcitabine (FDR)	6.0	--	0.88**	0.73 – 1.05
		Gemcitabine (FDR) + Oxaliplatin	5.9			
Heinemann	2006	Gemcitabine	6.0	0.15	0.80	0.59 – 1.08
		Gemcitabine + Cisplatin	7.5			
Colucci	2002	Gemcitabine	5.0	0.48	0.87	0.58 – 1.29
		Gemcitabine + Cisplatin	7.5			
Viret	2004	Gemcitabine	6.7	0.73	0.92	0.59 – 1.45
		Gemcitabine + Cisplatin	8.0			

FDR = fixed dose rate;

* FDR Gemcitabine versus Gemcitabine

** GEMOX versus Gemcitabine

### Gemcitabine plus fluoropyrimidine versus single-agent gemcitabine

The combination of gemcitabine with a fluoropyrimidine was tested in six randomized trials including 1813 patients in total (912 in the control arm and 901 in the combination arm) (Tables 2, 6, 7). Three trials used 5-FU and further three used capecitabine as a combination partner. A significant impact on ORR was observed only in the study by Cunningham and coworkers [13], on PFS in the trial reported by Berlin and coworkers [8]. The comparative analysis of OS did not show a benefit for the combination of gemcitabine with infusional 5-FU [9,10], while there was a trend towards an improved survival when gemcitabine was combined with bolus 5-FU (HR = 0.82, p = 0.09) [8].

**Table 6:** Overall response rate and PFS in 6 randomized trials comparing gemcitabine to gemcitabine plus fluoropyrimidine

**Reference**	**Year**	**n**	**Treatment regimen**	**ORR (%)**	**p**	**Median PFS/TTP (mo)**	**p**
Berlin	2002	162	Gemcitabine	5.6	--	2.2	
		160	Gemcitabine + 5-FU (bolus)	6.9		3.4	0.022
Riess	2005	238	Gemcitabine	7.2	--	3.5	0.44
		235	Gemcitabine + 5-FU (infusional)	4.8		3.5	
DiCostanzo	2005	48	Gemcitabine	8	--	3.5	--
		43	Gemcitabine + 5-FU (CI)	11		4.5	
Scheithauer	2003	42	Gemcitabine	14	--	4.0	--
		41	Gemcitabine + Capecitabine	17		5.1	
Herrmann	2005	159	Gemcitabine	7.9	--	4.0	0.207
		160	Gemcitabine + Capecitabine	10.1		4.8	
Cunningham	2005	266	Gemcitabine	7.1	0.008	na	na
		267	Gemcitabine + Capecitabine	14.2		na	

ORR = overall response rate; PFS = progression-free survival; TTP = time-to-progression; na = data not available; CI = continuous infusion; 5-FU = 5-fluorouracil;

**Table 7:** Survival in trials comparing gemcitabine to gemcitabine plus fluoropyrimidine

**Reference**	**Year**	**Treatment regimen**	**Median survival (mo)**	**p**	**HR**	**95% CI**
Berlin	2002	Gemcitabine	5.4	0.09	0.82	0.65 – 1.03
		Gemcitabine + 5-FU (bolus)	6.7			
Riess	2005	Gemcitabine	6.2	0.68	1.04	0.86 – 1.25
		Gemcitabine + 5-FU (infusional)	5.9			
DiCostanzo	2005	Gemcitabine	7.8	--	na	na
		Gemcitabine + 5-FU (CI)	7.5			
Scheithauer	2003	Gemcitabine	8.2	--	0.82	0.50 – 1.35
		Gemcitabine + Capecitabine	9.5			
Herrmann	2005	Gemcitabine	7.3	0.314	0.89	0.70 – 1.12
		Gemcitabine + Capecitabine	8.4			
Cunningham	2005	Gemcitabine	6.0	0.026	0.79	0.65 – 0.97
		Gemcitabine + Capecitabine	7.4			

5-FU: 5-fluorouracil; CI = continuous infusion; na = data not available;

The combined analysis of all six studies provides evidence that a moderate, but significant prolongation of survival can be expected from the combination of gemcitabine with a fluoropyrimidine (HR = 0.90, p = 0.03). The combination of gemcitabine with capecitabine caused a significant prolongation of OS in one trial (Cunningham), while this was not the case in two other trials [11,12]. Nevertheless, the effect of the gemcitabine/capecitabine combination on survival appears to show greater consistency as compared to the 5-FU combinations. This is reflected by a pooled HR of 0.83 (p = 0.01) in favour of the gemcitabine/capecitabine combination observed in three trials [11-13].

### Gemcitabine plus other cytotoxic agent versus single-agent gemcitabine

A total of 1404 patients were included into the remaining four randomized trials, formally combined to the group "other", which evaluated the combination of gemcitabine with the multitarget antifolate pemetrexed or the topoisomerase inhibitors irinotecan or exatecan (Tables 3, 8, 9). Only ORR was significantly improved by the combination of gemcitabine with pemetrexed and irinotecan [14,15]. The combined analysis of OS revealed a HR of 0.99 (p = 0.80) and failed to provide any indication for a benefit from combination chemotherapy including these agents.

**Table 8:** Overall response rate and PFS in 4 randomized trials comparing gemcitabine to gemcitabine plus other cytotoxic agent

**Reference**	**Year**	**n**	**Treatment regimen**	**ORR (%)**	**p**	**Median PFS/TTP (mo)**	**p**
Oettle	2005	282	Gemcitabine	7.1	0.004	3.3	0.111
		283	Gemcitabine + Pemetrexed	14.8		3.9	
Rocha Lima	2004	180	Gemcitabine	4.4	< 0.001	3.0	0.352
		180	Gemcitabine + Irinotecan	16.1		3.5	
Stathopoulos	2006	70	Gemcitabine	10	0.387	2.9	0.795
		60	Gemcitabine + Irinotecan	15		2.8	
O'Reilly	2004	174	Gemcitabine	7.1	--	3.8	0.22
		175	Gemcitabine + Exatecan	8.2		4.1	

ORR = overall response rate; PFS = progression-free survival; TTP = time-to-progression;

**Table 9:** Survival in trials comparing gemcitabine to gemcitabine plus other cytotoxic agent

**Reference**	**Year**	**Treatment regimen**	**Median survival (mo)**	**P**	**HR**	**95% CI**
Oettle	2005	Gemcitabine	6.3	0.848	0.98	0.82 – 1.18
		Gemcitabine + Pemetrexed	6.2			
Rocha Lima	2004	Gemcitabine	6.6	0.789	1.04	0.84 – 1.30
		Gemcitabine + Irinotecan	6.3			
Stathopoulos	2006	Gemcitabine	6.5	0.970	na	na
		Gemcitabine + Irinotecan	6.4			
O'Reilly	2004	Gemcitabine	6.2	0.52	0.93	0.74 – 1.17
		Gemcitabine + Exatecan	6.7			

na = data not available;

### Total analysis of 15 randomized trials

**Figure 1:**
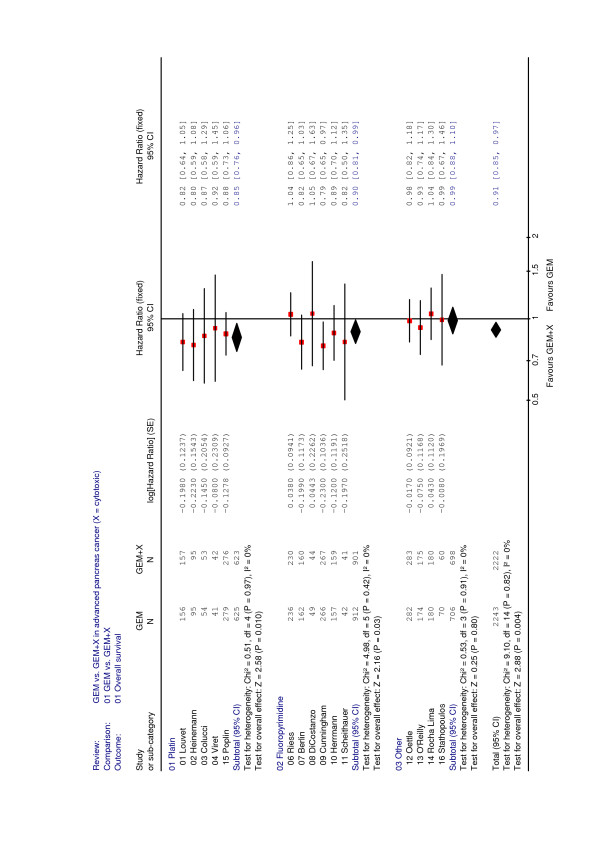
Meta-analysis for combination chemotherapy in advanced pancreatic cancer – overall survival with regard to combination partner (platinum analog, fluoropyrimidine or other) for gemcitabine.

The total analysis of 15 randomized trials involving 4465 patients demonstrates a moderate, but significant benefit from gemcitabine-based combination chemotherapy when compared to gemcitabine alone (HR = 0.91, p = 0.004) (Table 10). Clearly this benefit is essentially derived from combinations of gemcitabine with either platinum analogs or fluoropyrimidines (Figure 1, fixed effect model). Both for the total and the subgroup analyses the appropriate tests do not reveal any major heterogeneity between the trial results. Accordingly, the application of random effect models gave results not deviating at all from those presented in the forest plot.

**Table 10:** Meta-analysis – Survival in 15 trials comparing gemcitabine to gemcitabine + cytotoxic agent

**Groups**	**n trials**	**n patients**	**HR**	**p**	**95% CI**
Gemcitabine versus Gemcitabine + Platinum Analog	5	1248	0.85	0.010	0.76 – 0.96
Gemcitabine versus Gemcitabine + Fluoropyrimidine	6	1813	0.90	0.03	0.81 – 0.99
Gemcitabine versus Gemcitabine + other cytotoxic agent	4	1404	0.99	0.80	0.88 – 1.10

**Total**	**15**	**4465**	**0.91**	**0.004**	**0.85 – 0.97**

### Subgroup analysis of performance status

**Figure 2:**
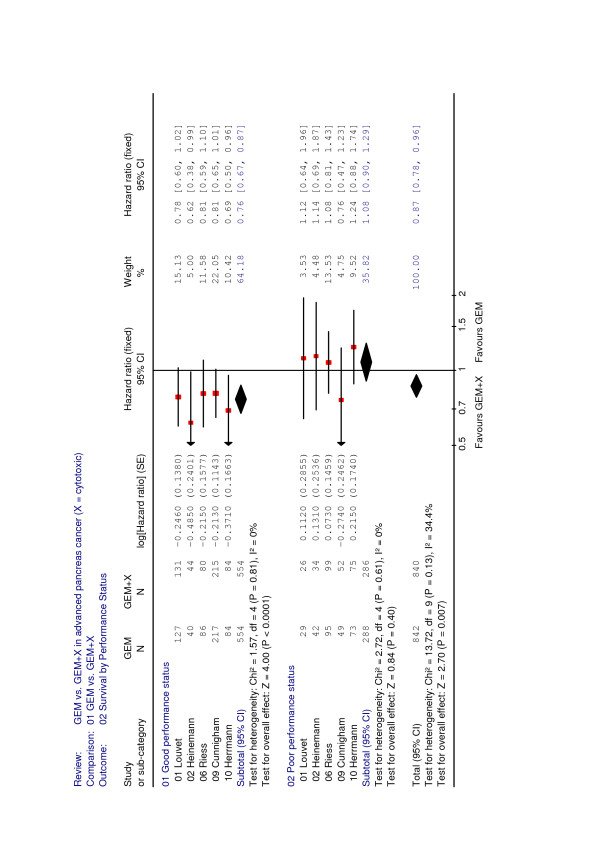
Meta-analysis for combination chemotherapy in advanced pancreatic cancer – overall survival with regard to performance status.

A planned subgroup analysis divided patients into a good performance status (KPS = 90–100%, ECOG 0–1) or a poor performance status cohort (KPS 60–80%, ECOG 2). Data from five randomized trials including 1682 patients (1108 good performance status versus 574 poor performance status) provided evidence on treatment outcome in the two subgroups [3,5,9,12,13]. The remaining 10 randomized trials (representing 2783 patients) did not report subgroup data based on performance status. A highly significant benefit from combination chemotherapy was observed in patients with a good performance status (HR = 0.76, p < 0.001). By contrast, patients with a poor performance status did not appear to benefit from combination chemotherapy (HR = 1.08, p = 0.40) (Figure 2).

## Discussion and Conclusion

Pancreatic cancer is a highly malignant disease, and survival is expected to be short in advanced disease. Once treatment has been initiated, response evaluation by imaging is difficult and tumor response is not regarded as a reliable parameter of treatment efficacy. It therefore appears that OS should be evaluated as a primary endpoint when different treatment options and therapeutic regimens are compared. In view of the rather short course of the disease first-line therapy is expected to have the greatest impact on OS. Accordingly, the present meta-analysis chose to evaluate 15 randomized trials based on the available survival data only.

The starting point of this analysis has been the perception that single-agent gemcitabine as the present standard of care is only moderately active in metastatic pancreatic cancer and allows a median OS of only 5–8 months in randomized trials. In view of the manifold trials investigating gemcitabine-based combination therapies only two studies stand out which reported a significant improvement of survival in favour of the combination therapy [13,23]. In both trials patient numbers exceeded 500, and the hazard ratios achieved in favour of the combination were nearly identical: HR = 0.80 (p = 0.026) for gemcitabine plus capecitabine [13], and HR = 0.81 (p = 0.025) for gemcitabine plus erlotinib [18].

This meta-analysis evaluated the 15 available trials comparing gemcitabine versus gemcitabine plus one other chemotherapy drug excluding combined therapy with targeted agents. When all 15 trials (4465 patients) are taken together a highly significant (p = 0.004) advantage of survival is obtained in favour of combination therapy. However, the gain in survival time is slim (HR = 0.91; 95% CI 0.85 – 0.97) and clinical relevance remains moderate.

In a subsequent step, trials were grouped according to the combination partner and separate analyses were performed for combinations with either platinum analogs, fluoropyrimidines or "other" agents (Table 10). This analysis indicated that the combination of gemcitabine plus a platinum analog (cisplatin or oxaliplatin) was significantly superior to gemcitabine alone inducing a HR of 0.85 (p = 0.01) with a low heterogeneity of results (p = 0.97). However, one must keep in mind that the study of Louvet and colleagues as well as the E 6201 study by Poplin and co-workers used a FDR gemcitabine application [3,4] in the combination arm (i.e., gemcitabine in the GemOx arm was not given as a standard 30-minute infusion [5-7], but at a FDR infusion of 10 mg/m^2^/min). Also the combination of gemcitabine with a fluoropyrimidine induced a significant survival benefit (HR = 0.90, p = 0.03). The somewhat heterogeneous result (p = 0.42) in this group of trials was essentially due to the more inconsistent survival data obtained by the trials using 5-FU as a combination partner. By contrast, when only the three trials using capecitabine as a combination partner were analysed together, a HR of 0.83 (p = 0.01) was obtained.

This leads to the conclusion that the combination of gemcitabine with either a platinum analog or capecitabine may allow a clinically relevant prolongation of survival supported by hazard ratios in the range of 0.83 – 0.85. Compared to these positive results, combinations of gemcitabine with either pemetrexed or topoisomerase I inhibitors (irinotecan or exatecan) did not have any effect on survival (HR = 0.99) and consequently have no place in clinical practice.

To date, five meta-analyses evaluating radiotherapy and chemotherapy in advanced, non-resectable pancreatic cancer have been published [24-28]. These analyses showed that chemotherapy is able to prolong survival (compared to best supportive care only) in patients with advanced pancreatic cancer [24,26], and there is also evidence that gemcitabine-based combination chemotherapy may be superior to single-agent gemcitabine regarding overall survival [25,26,28]. In accordance to our data, the most promising survival advantage was observed when gemcitabine was combined with either a platinum compound or capecitabine: Sultana and colleagues in their meta-analysis for example reported a HR of 0.85 (95% CI 0.74 – 0.96, p = 0.01) for the addition of cisplatin or oxaliplatin to standard gemcitabine and a HR of 0.83 (95% CI 0.72 – 0.96, p = 0.01) for the addition of capecitabine to single-agent gemcitabine, respectively [26]. Further meta-analytic data on treatment efficacy (e. g. time-to-progression, progression-free survival, response rate) and toxicity variables have also been reported [25,27], however such an analysis was not the intent of our investigations.

In a further step of our meta-analysis, those trials were identified and evaluated in which survival data were reported in patient subgroups with a defined performance status. Data from 1682 patients only were available for this pre-defined subgroup analysis, representing about 38% of all patients (4465) from this meta-analysis. Thus, these results should be regarded carefully as a possible outcome reporting bias can not be excluded. Storniolo and coworkers had previously demonstrated that single-agent treatment with gemcitabine induced a median survival of 5.5 months in patients with a KPS ≥ 70%, while patients with a KPS < 70% did not appear to profit from therapy (median OS = 2.4 months) [29]. Likewise, single randomized studies have indicated that a benefit from combination chemotherapy can only be expected in patients with a good performance status [30]. The present meta-analysis of five trials indicates that combination chemotherapy induces its greatest benefit in patients with a good performance status [3,5,9,12,13]. In these patients (ECOG 0–1 or KPS = 90–100%), a combination of gemcitabine with platinum analogs or fluoropyrimidines induced a statistically significant and also clinically relevant HR of 0.76 (p < 0.0001). By contrast, patients with a poor KPS of 60–80% rather seem to have no survival advantage from the more intensive combination chemotherapy (HR = 1.08).

In conclusion, the subgroup analysis of five large randomized trials provides a possible rationale in favour of combination chemotherapy when applied in good performance status patients who can tolerate prolonged intensive therapy. However, post-hoc subgroup analyses from single randomized trials can only be regarded as hypothesis-generating, and even if there is increasing evidence for an important prognostic role of performance status, a prospective evaluation of this (clinically relevant) issue is strongly recommended for future clinical trials in advanced pancreatic cancer. A re-evaluation of performance status data from all the 15 trials included in this meta-analysis – even perhaps based on individual patient data – would be another promising approach to overcome the limitations of a possible outcome reporting bias.

This meta-analysis was focused on gemcitabine-based chemotherapy combinations and excluded combinations with targeted agents. The results therefore pertain only to the referred chemotherapy doublets. Randomized trials comparing gemcitabine versus gemcitabine plus metalloproteinase inhibitors, tipifarnib or bevacizumab did not show a significant survival benefit [31-34]. More promising results were obtained from inhibition of the epidermal growth factor receptor (EGFR) by the oral tyrosinekinase inhibitor erlotinib. The combination of gemcitabine with erlotinib induced a significant improvement of PFS and OS when compared to gemcitabine alone [23]. However, preliminary data from a randomized trial investigating the EGFR-directed antibody cetuximab as a combination partner (SWOG S0205) did not show a significant survival benefit for gemcitabine plus cetuximab compared to gemcitabine monotherapy [35].

The question needs to be asked if the results of this meta-analysis have an impact on the design of future trials performed in pancreatic cancer. In conclusion, the following statements can be made:

1. One might consider separate treatment strategies for patients with good and poor performance status in future clinical trials.

2. It has become clear that combination chemotherapy may be a valuable tool to improve treatment efficacy in patients with a good performance status. Further prospective exploration of intensive treatment is needed specifically in this patient group.

3. Patients with a poor performance status possibly have no further benefit from combination chemotherapy and thus should perhaps rather receive single-agent gemcitabine. They also should be candidates for new investigational treatment approaches.

## Competing interests

Volker Heinemann: Received research funding and honoraria for consultant activities from Lilly Germany. Stefan Boeck: Received honoraria for scientific presentations from Lilly Germany. The remaining authors have no competing interests to declare.

## Authors' contributions

VH, SB, AH, RL and CL were responsible for the design of this meta-analysis, for data acquisition and interpretation of data. VH and AH did the statistical analyses. VH, SB, AH, RL and CL have been involved in drafting the manuscript and have given final approval of the current version of the manuscript.

## Pre-publication history

The pre-publication history for this paper can be accessed here:


